# Relationship between area-level socioeconomic characteristics and outdoor NO_2_ concentrations in rural and urban areas of northern Spain

**DOI:** 10.1186/1471-2458-13-71

**Published:** 2013-01-25

**Authors:** Ana Fernández-Somoano, Gerard Hoek, Adonina Tardon

**Affiliations:** 1 Spanish Consortium for Research on Epidemiology and Public Health (CIBERESP), Instituto de Salud Carlos III, C/Melchor Fernández Almagro, 3-5. Pabellón 6, planta baja, 28029, Madrid, Spain; 2 Preventive Medicine and Public Health, University of Oviedo, c/Julián Clavería, 33006, Oviedo, Spain; 3 Institute for Risk Assessment Sciences, Environmental Epidemiology Division, Universiteit Utrecht, 3508 TD, Utrecht, Netherlands

**Keywords:** Air pollution, Socioeconomic factors, Education, Nitrogen dioxide

## Abstract

**Background:**

Socioeconomic variables are associated with mortality and morbidity in a variety of diseases at both the individual and neighborhood level. Investigating whether low socioeconomic status populations are exposed to higher air pollution has been an important objective for the scientific community during the last decade. The goal of this study was to analyze the associations between outdoor nitrogen dioxide (NO_2_) concentrations in an area of Asturias (Spain) and two socioeconomic indexes—one based on occupation and the other on educational level—at the census-tract level.

**Methods:**

A map of NO_2_ concentration was obtained from a land-use regression model. To obtain a census-tract average value, NO_2_ was estimated at the centroids of all 50 × 50 m grids within a census tract. Standard socioeconomic variables were used from the Census of Population and Housing 2001. We analyzed the association between NO_2_ concentration and socioeconomic indicators for the entire area and stratified for more urban and more rural areas.

**Results:**

A positive linear relationship was found between the levels of education and NO_2_ exposure in the urban area and the overall study area, but no association was found in the rural area. A positive association between socioeconomic index based upon occupation and NO_2_ concentration was found in urban areas; however, this association was reversed in the rural and overall study areas.

**Conclusions:**

The strength and direction of the association between socioeconomic status and NO_2_ concentration depended on the socioeconomic indicator used and the characteristics of the study area (urban, rural). More research is needed with different scenarios to clarify the uncertain relationship among socioeconomic indexes, particularly in non-urban areas, where little has been documented on this topic.

## Background

Air pollution is a major environmental risk factor, affecting the health of the population. Exposure to air pollution may vary according to different socioeconomic and demographic conditions [1-5]. Thus, interest in socioeconomic factors has recently increased in environmental epidemiology and public health research [3,6,7]. It has been well established that low levels of education and low income are associated with higher mortality and morbidity [3]. Research has indicated that socioeconomic factors at the individual and neighborhood level may influence individual health status [3,6,7]. In epidemiological studies on the health effect of air pollution, socioeconomic variables may act as a confounding factor but also as effect modifiers. O’Neill et al. in 2003 [3] provided three possible explanations for an interaction between socioeconomic variables and air pollution in terms of health effects, including increased exposure, increased susceptibility to air pollution exposure, and increased occurrence of co-morbidity in more deprived individuals/areas. Some studies have shown that socioeconomic conditions can modify the effect of air pollution on mortality [1,8-14]; other studies have indicated that socioeconomic conditions have a confounding effect with respect to air pollution and mortality [15,16].

Investigating whether populations with low socioeconomic status are more exposed to air pollution has been an important objective for the scientific community during the last decade [3,17,18]. Several studies have found that disadvantaged groups experience the worst environmental conditions [19-23]. On the other hand, some recent studies have identified greater exposure in areas of higher socioeconomic status [8,24-26], which indicates that the relationship between air pollution and socioeconomic characteristics may differ from place to place. Further research is needed to understand the complexity of these associations. Some differences in the results of these studies may be due to methodological differences, e.g., in the definition of geographic areas, the socioeconomic characteristics examined, and the level of detail of exposure assessment. In addition, there are limits in making generalizations from studies conducted at a particular site [27]; the direction and magnitude of the various associations may differ depending on the size and scale of the study area [20,24]. Most of the studies investigating this issue have been carried out in large cities but not in rural or semiurban areas, where distributions and population characteristics may be different. There is also a wide range of variables used as socioeconomic indicators [17]. The selection of the index may be important when looking for a relationship between socioeconomic characteristics and air pollution exposure.

Moreover, exposure to ambient air pollutants, especially to particulates, has been consistently associated with mortality and morbidity. Vehicle exhausts are an important source of particulates; models of outdoor nitrogen dioxide (NO_2_) exposure have been used extensively as to characterize exposure to traffic-related air pollutants [28,29], particularly when assessing medium- to long-term exposure [30].

Our hypothesis is that there is a relationship between socioeconomic status and pollution. Knowledge of this association is important for population risk assessment, as it is well established that baseline morbidity and mortality rates differ with socioeconomic status. If exposure also differs by socio-economic status, assessments need to incorporate socio-economic variables.

The identification of geographic areas with greater air pollution exposure and worse socioeconomic level would facilitate the implementation of interventions and policies to tackle inqualities in the population. Small-area analysis offers the chance to gain a deeper understanding of geographic patterns.

The goal of this study was to analyze the association between fine-scale spatial variation of outdoor NO_2_ concentrations in an industrial area of Asturias (Spain) and two socioeconomic indices—one based on occupation and activity, and the other based on educational level—at the census-tract level. Specific objectives were further to investigate whether there were differences in these associations between urban and more rural areas.

## Methods

### Study population

We performed a cross-sectional ecological study in which the units of analysis were census tracts—the smallest spatial level of disaggregation for which socioeconomic census data is available. A census tract is a partition of a municipality that is typically defined by easily identifiable boundaries, including natural features as well as features such as buildings, major roads, and land use. A census tract has approximately 1,000 to 2,000 residents, except when a municipality has a smaller population. At the time of the Population and Housing Statistics 2001 [31], the total number of census tracts in the study area was 138. The study population consisted of residents (n = 154,918 inhabitants) in sanitary area III of Asturias, having an area of 483 km^2^. Sanitary area III consists of nine municipalities: Avilés (the third-biggest city in Asturias in terms of economy and population, with 83,517 inhabitants and a population density of 3,115 per km^2^ in 2008) and the nearby districts of Gozón, Castrillón, Corvera de Asturias, Muros de Nalón, Soto del Barco, Cudillero, Pravia and Illas. This area was selected because a number of different epidemiological studies are being carried out in collaboration with the reference hospital, San Agustin, which is located in Avilés; this studies include the multicenter INMA (INfancia y Medio Ambiente [Environment and Childhood]) project [32,33]. Aluminum, steel, glass, and chemical industries as well as road traffic are the principal sources of air pollution in this area.

The research protocol for this study was approved by the ethics committee of the center involved.

### Socioeconomic status and air pollution measurements

We used the standard socioeconomic variables of the Population and Housing Census 2001, which was published in 2004 by the National Statistical Institute in Spain (INE) [31]. This census provides municipality information for the whole country. We used information at the census-tract level.

#### Mean socioeconomic index

The socioeconomic status index was derived from a standard Spanish classification based on occupation and activity (http://www.ine.es/censo/en/glosario.html). Additional file 1 lists the grades that are assigned to different occupations. The grades range from 0 (unemployed) to 3 (manager). The socioeconomic status index is calculated as the arithmetic mean of the grades of all members of a household. The index thus depends on age distribution, which was taken into account in the data analysis.

#### Education

Education was classified on a scale ranging from 0 (illiterate) to 4.5 (PhD level). Additional file 2 provides the exact definitions. It was considered that a person had reached a certain level of education when he or she has completed and passed all courses at that level and was therefore able to obtain the corresponding diploma. The household education level was defined as the arithmetic mean educational level of the family members. Thus, the educational level also depended on age distribution.

#### NO_2_ levels

The NO_2_ concentration map was obtained from a land-use regression (LUR) model [34]. Briefly, NO_2_ (μg/m^3^) was measured simultaneously at 67 sampling points covering the study area during two 1-week periods (in June and November) in 2005. These short-term measurements are a valid method for characterizing spatial contrasts though not absolute concentration levels. Then, a linear regression model was fitted using geographic data (land use, roads, altitude and distance to industrial facilities). The final model (R^2^ = 0.521) included agricultural and forest land cover factors within a 300-m buffer as well as altitude and distance to the nearest road (any road) as predictor variables. All regression slopes of the model were negative, which was consistent with knowledge of emissions and the dispersion of traffic-related air pollution.

To obtain a census-tract average value, NO_2_ (μg/m^3^) was estimated at the centroids of all 50 × 50 m grids within a census tract. Then, the average of all NO_2_ estimates within a census tract was used for further analysis.

### Statistical analysis

We determined NO_2_ levels in addition to the socioeconomic index and education across the census tracts and calculated the correlations among them. The association among those variables was analyzed using Spearman’s rank correlation in order to determine the correlation when the relation was not linear.

We also stratified for census tracts with less than 50% urban land (all municipalities except Avilés and 5 census tracts of this township) and those with at least 50% urban land (the remaining census tracts of Avilés); here, we took urban as a habitable area with over 10,000 inhabitants. Since the indexes used are age-dependent, we also adjusted for age distribution at the census-tract level. We used the percentage of potential working population as adjustment factors, considering these to be people aged 16–64 years.

We categorized study variables based on natural groupings inherent in the data using the Jenks optimization method (also called the Jenks natural breaks classification method), which is a data-classification method designed to determine the best arrangement of values in different classes. This is achieved by seeking to minimize each class’s average deviation from the class mean while maximizing each class’s deviation from the means of the other groups. In other words, the method seeks to reduce the variance within classes and maximize the variance between classes [35,36].

Spatial autocorrelation of the distributions of NO_2_ levels and of the socioeconomic indexes was estimated by calculating the Moran index (I) [37]. This coefficient varies between −1 for a negative spatial autocorrelation and +1 for a positive spatial autocorrelation. Values of Moran’s I are assessed by a test statistic (the Moran’s I standard deviate) which indicates the statistical significance of the spatial autocorrelation.

As the Moran index showed a statistically significant spatial autocorrelation in the residuals of a linear regression model, a spatial regression model was applied. We selected the best simultaneous autoregressive (SAR) model specification with the Lagrange multiplier test statistics developed by Anselin et al. [38,39], which led us to choose an SAR lag model that takes the form:

$$\begin{array}{l} y\;=\;\text{ρ Wy}\;+\;\text{SE}\;\text{index β}\;+\;\text{Education γ}\\ \;+\;\varepsilon ;\;\mathrm{with}\;\varepsilon \approx \mathrm{iid}\;\left(0;\sigma^{2}\right) \end{array}$$

Where y corresponds to NO_2_ levels, *β* to the regression coefficient associated with the socioeconomic index, *γ* to the regression coefficient associated with the educational level, and *ε* to model residuals assumed to be independently and identically distributed (i.i.d.). W corresponds to a spatial weight matrix that defined the notion of neighborhood between geographic units, and *ρ* to a spatial autoregressive parameter that estimates the scale of interactions between the observations of the dependent variable. The SAR lag model is similar to a linear regression model in which a spatially lagged dependent variable Wy is introduced to control for spatial autocorrelation [40].

Statistical analyses were performed using SPSS (Statistical Package for the Social Sciences) 15.0 for Windows, R (The R Foundation for Statistical Computing) 2.15.2 and OpenGeoDa (GeoDa Center for Geospatial Analysis and Computation and Arizona Board of Regents) 0.9.8.14. Maps were drawn with ArcGIS 10 (ESRI, Redlands, CA, USA).

## Results

Table 1 presents the distribution of the population and socioeconomic characteristics by census tract both for areas with less than 50% urban land and those with at least 50% urban land. Urban areas accounted for a greater percentage of unemployed people but a smaller percentage of low-educated individuals.

**Table 1:** Population distribution and socioeconomic characteristics across the census tracts

		***n***	***Mean***	***SD***	***Median***	***Min***	***Max***
**Family housing residents**	**Urban <50%**	70	1096	386	1059	536	2047
	**Urban ≥50%**	68	1150	387	1129	504	2013
	**All**	138	1123	386	1096	504	2047
**% 16–64 years**	**Urban <50%**	70	66.85	6.48	66.19	52.81	79.19
	**Urban ≥50%**	68	68.16	5.30	68.89	51.53	77.70
	**All**	138	67.49	5.94	67.88	51.53	79.19
**% Unemployment**	**Urban <50%**	70	6.34	1.38	6.43	1.87	9.17
	**Urban ≥50%**	68	7.81	1.95	7.53	4.83	14.50
	**All**	138	7.06	1.83	6.90	1.87	14.50
**% Manual workers**	**Urban <50%**	70	4.77	1.81	4.45	1.25	9.17
	**Urban ≥50%**	68	4.56	1.96	4.52	0.69	11.55
	**All**	138	4.67	1.88	4.50	0.69	11.55
**% low education**	**Urban <50%**	70	48.03	13.16	48.30	18.26	87.74
	**Urban ≥50%**	68	42.54	10.12	40.30	23.86	63.28
	**All**	138	45.33	12.03	44.92	18.26	87.74

The average number of inhabitants per census tract was 1123 (standard deviation 386; median 1096). For census tracts with less than 50% urban area, the average was 1096 (standard deviation 386; median 1059); for census tracts with at least 50% urban land, the average was 1150 (standard deviation 387; median 1129). Socioeconomic indexes—one based on occupation and activity, the other based on educational level—and mean NO_2_ levels (μg/m^3^) appear in Table 2.

**Table 2:** **Distribution of census tract level modeled NO**_**2 **_**concentration (μg/m**^**3**^**), socioeconomic index, and educational level**

		**n**	**Mean**	**S. D.**	**Min**	**P25**	**Median**	**P75**	**Max**
**NO**_**2**_	**Urban <50%**	70		12.92	5.57	3.23	8.37	11.84	17.33	25.67
	**Urban ≥50%**	68		23.26	4.22	10.38	22.10	24.18	25.70	30.49
	**All**	138		18.02	7.16	3.23	11.32	19.42	24.19	30.49
SE index* ^**a**^	**Urban <50%**	70		1.42	0.18	1.17	1.27	1.41	1.52	2.05
	**Urban ≥50%**	68		1.35	0.14	1.04	1.28	1.33	1.42	1.69
	**All**	138		1.38	0.16	1.04	1.27	1.34	1.48	2.05
Education* ^**b**^	**Urban <50%**	70		3.38	0.32	2.72	3.10	3.39	3.58	4.10
	**Urban ≥50%**	68		3.49	0.32	2.93	3.32	3.44	3.62	4.26
	**All**	138		3.43	0.32	2.72	3.21	3.40	3.61	4.26

^a^ SE index is based on occupation and activity (range 0–3);

^b^ Education is level of studies (range 0–4.5).

^*^ Age adjusted.

Concentrations of NO_2_ were clearly higher in mostly urban areas. Higher educational level but a lower socioeconomic index was found in urban areas. The average educational value of 3.4 recorded in the overall study area corresponds approximately to a higher grade of vocational training, an industrial master’s qualification or equivalent, an associate degree, architecture and engineering techniques, or having completed three approved courses toward degrees in the fields of engineering or architecture (Additional file 2). The average occupational index for all census tracts of about 1.4 corresponds to agricultural workers without employees and members of agricultural cooperatives (Additional file 1).

**Figure 1:**
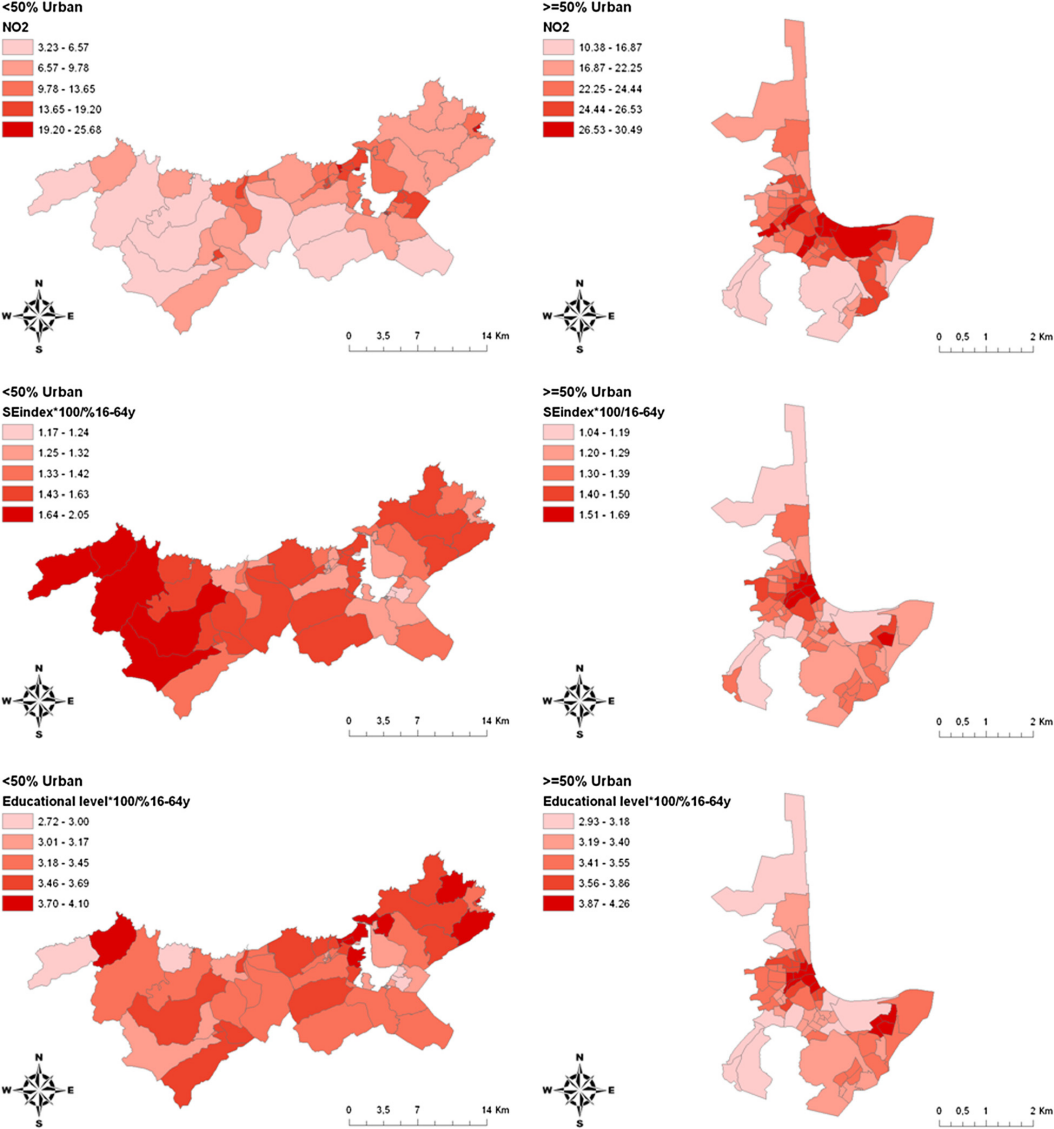
**Distribution maps for NO**_**2 **_**concentration (μg/m**^**3**^**), socioeconomic index (occupation), and education.** NOTE: The white area in left figure corresponds to right figure.

**Figure 2:**
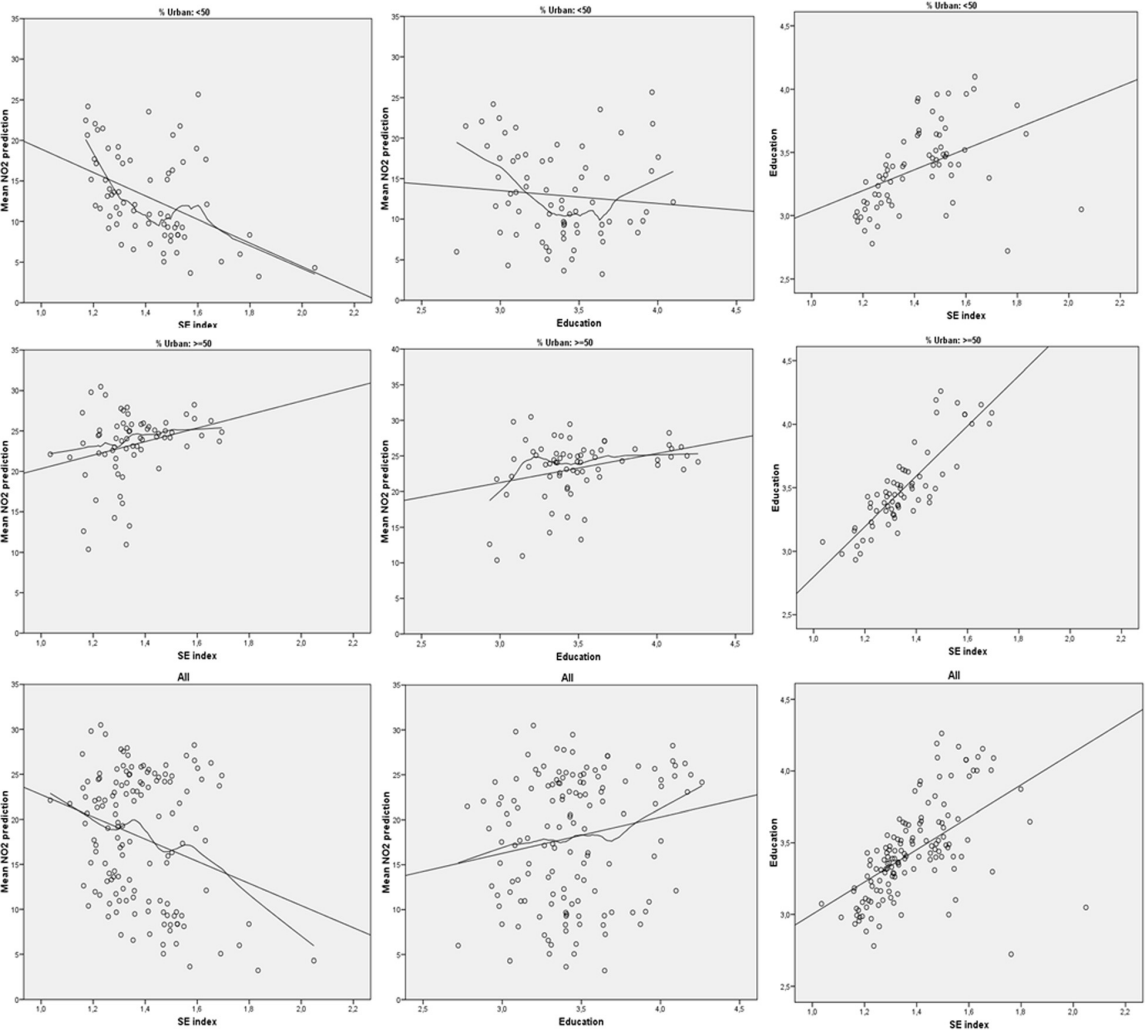
**Relationships among census-tract socioeconomic index (occupation), education and NO**_**2 **_**concentration (μg/m**^**3**^**).** NOTE: Loess and linear lines.

Figure 1 shows the spatial distribution of mean NO_2_ levels in the census tract in addition to the socioeconomic index and educational level for census tracts with less than 50% urban area (Figure 1a) and for those with at least 50% urban area (Figure 1b). It is notable that the three variables are positively correlated, particularly within the urban areas. The pattern of associations is clearer in the scatter plot (Figure 2) and the categorical analysis presented in Table 3.

**Table 3:** **Mean census-tract NO**_**2 **_**concentration (μg/m**^**3**^**) by socioeconomic index category (based on occupation) and educational level**

**Land**	SE index* ^**a**^	**NO**_**2**_			Education* ^**b**^	**NO**_**2**_		
		**n**	**Mean**	**S. D.**		**n**	**Mean**	**S. D.**
**Urban <50%**	**1.04-1.24**	11	18.71	4.31	**2.72-3.03**	11	17.14	6.11
	**1.25-1.34**	16	13.29	3.55	**3.04-3.24**	13	13.40	4.62
	**1.35-1.46**	10	11.69	4.81	**3.25-3.49**	25	10.01	3.97
	**1.47-1.65**	24	12.43	5.65	**3.50-3.82**	12	13.30	6.04
	**1.66-2.05**	5	5.39	1.94	**3.83-4.26**	8	15.27	6.14
**Urban ≥50%**	**1.04-1.24**	14	22.15	5.85	**2.72-3.03**	3	14.91	6.01
	**1.25-1.34**	23	21.95	4.87	**3.04-3.24**	10	23.89	5.62
	**1.35-1.46**	14	23.99	1.60	**3.25-3.49**	26	23.06	3.68
	**1.47-1.65**	10	25.35	1.58	**3.50-3.82**	15	23.23	3.89
	**1.66-2.05**	2	24.29	0.81	**3.83-4.26**	10	25.41	1.51
**All**	**1.04-1.24**	25	20.64	5.42	**2.72-3.03**	14	16.66	5.93
	**1.25-1.34**	39	18.40	6.11	**3.04-3.24**	23	17.96	7.27
	**1.35-1.46**	24	18.87	6.99	**3.25-3.49**	51	16.66	7.60
	**1.47-1.65**	34	16.23	7.66	**3.50-3.82**	27	18.82	6.99
	**1.66-2.05**	7	10.79	9.36	**3.83-4.26**	18	20.90	6.60

Cut points defined on natural groupings (methods).

^a^ SE index is based on occupation and activity (range 0–3);

^b^ Education is level of studies (range 0–4.5).

^*^ Age adjusted.

When performing linear regression we found strong spatial autocorrelation in the residuals (the Moran I test statistic for spatial autocorrelation applied to regression residuals was statistically significant). This suggested us the use of a spatial regression model where the spatial autoregressive (SAR) parameter (Rho) was highly statistically significant. There was no autocorrelation in the residuals of the spatial regression model. We found strong negative associations between NO_2_ levels and the socioeconomic index in *all census tracts* and *census tracts with less than 50% urban land* (Table 4). Pollution levels were statistically significant lower in census tracts with higher socioeconomic index. No association was found in *mainly urban census tracts*. NO_2_ was not consistently related to educational level in all census tracts and the less urban census tracts. We found the only statistically significant association with NO_2_ levels in category 5 of education in *all census tracts*, being people with higher education those exposed to higher levels of air pollution. In the urban census tracts, higher education was associated with higher NO_2_. Models with both socio-economic variables in the model are difficult to interpret in the urban census tracts because of the high correlation (Spearman R = 0.81).

**Table 4:** **Associations between NO**_**2 **_**(μg/m**^**3**^**) and socioeconomic indices**

**Land**		***ρ* Wy + SEindex**^**a **^***β*+Education**^**b **^***γ***			***ρ* Wy + SEindex *β***			***ρ* Wy + Education *γ***		
		**Coeff.**	**SE**	**p**	**Coeff.**	**SE**	**p**	**Coeff.**	**SE**	**p**
**Urban <50%**	**Intercept**	11.01	2.16	<0.001	8.59	1.95	<0.001	6.33	1.72	<0.001
	**SEindex**_**1.25–1.34**_	−2.44	1.51	0.105	−3.22	1.43	0.024			
	**SEindex**_**1.35–1.46**_	−4.89	1.99	0.014	−3.67	1.65	0.026			
	**SEindex**_**1.47–1.65**_	−4.74	1.72	0.006	−3.84	1.39	0.006			
	**SEindex**_**1.66–2.05**_	−9.15	2.25	<0.001	−7.50	2.20	<0.001			
	**Education **_**3.04–3.24**_	−1.25	1.52	0.410				−1.50	1.50	0.318
	**Education **_**3.25–3.49**_	−2.55	1.54	0.098				−3.99	1.35	0.003
	**Education **_**3.50–3.82**_	1.35	1.82	0.456				−1.28	1.51	0.395
	**Education **_**3.83–4.26**_	2.58	1.95	0.186				−0.73	1.69	0.665
		Rho = 0.50 (p-value <0.001)			Rho = 0.61 (p-value <0.001)			Rho = 0.69 (p-value <0.001)		
		Residual I p-value = 0.423			Residual I p-value = 0.098			Residual I p-value = 0.294		
		AIC = 395.52			Residual I p-value = 0.098 AIC = 402.50			AIC = 403.83		
		(AIC for linear model = 407.44)			(AIC for linear model = 422.24)			(AIC for linear model = 433.32)		
**Urban ≥50%**	**Intercept**	2.89	2.05	0.160	4.10	1.76	0.020	3.23	2.08	0.121
	**SEindex**_**1.25–1.34**_	1.36	1.09	0.211	1.05	0.80	0.194			
	**SEindex**_**1.35–1.46**_	1.31	1.29	0.310	0.70	0.90	0.438			
	**SEindex**_**1.47–1.65**_	2.04	1.68	0.226	1.90	0.98	0.053			
	**SEindex**_**1.66–2.05**_	0.75	2.47	0.760	0.83	0.85	0.653			
	**Education **_**3.04–3.24**_	2.90	1.63	0.076				3.34	1.66	0.045
	**Education **_**3.25–3.49**_	1.59	1.78	0.371				2.94	1.55	0.059
	**Education **_**3.50–3.82**_	1.20	1.95	0.540				2.78	1.62	0.086
	**Education **_**3.83–4.26**_	2.11	2.30	0.360				4.03	1.68	0.016
		Rho = 0.76 (p-value <0.001)			Rho = 0.79 (p-value <0.001)			Rho = 0.73 (p-value <0.001)		
		Residual I p-value = 0.180			Residual I p-value = 0.705			Residual I p-value = 0.111		
		AIC = 346.76			AIC = 342.83			AIC = 340.99		
		(AIC for linear model = 387.44)			(AIC for linear model = 393.5)			(AIC for linear model = 382.99)		
**All**	**Intercept**	4.50	1.32	<0.001	3.92	1.10	<0.001	2.81	1.21	0.020
	**SEindex**_**1.25–1.34**_	−1.76	0.97	0.070	−1.23	0.85	0.152			
	**SEindex**_**1.35–1.46**_	−2.70	1.23	0.029	−1.04	0.97	0.282			
	**SEindex**_**1.47–1.65**_	−3.64	1.18	0.002	−1.56	0.89	0.080			
	**SEindex**_**1.66–2.05**_	−5.49	1.66	0.001	−3.39	1.50	0.024			
	**Education **_**3.04–3.24**_	0.06	1.17	0.960				−0.29	1.17	0.803
	**Education **_**3.25–3.49**_	0.61	1.18	0.608				−0.82	1.04	0.429
	**Education **_**3.50–3.82**_	2.30	1.36	0.090				0.14	1.13	0.899
	**Education **_**3.83–4.26**_	3.59	1.48	0.015				0.49	1.22	0.687
		Rho = 0.83 (p-value <0.001)			Rho = 0.87 (p-value <0.001)			Rho = 0.88 (p-value <0.001)		
		Residual I p-value = 0.611			Residual I p-value = 0.552			Residual I p-value = 0.552		
		AIC = 777.53			AIC = 779.59			AIC = 782.96		
		(AIC for linear model = 906.94)			(AIC for linear model = 929.91)			(AIC for linear model = 940.67)		

^a^ Reference category 1.04–1.24; ^b^ Reference category 2.72–3.03.

## Discussion

At the census tract level, we examined the relationship between outdoor concentrations of NO_2_ and socioeconomic status in an area of northern Spain. Outdoor concentrations of NO_2_ are higher for higher level of education and with higher socioeconomic index based on occupation in census tracts with over 50% urban area. By contrast, in census tracts in more rural areas, we found higher NO_2_ concentrations with a lower socioeconomic index and no relationship with the mean educational level.

The strength of the association with outdoor NO_2_ concentration was different between the socioeconomic indicator based on occupational status and that based on education. The need for a careful definition of socioeconomic variables has been identified in previous studies as an important issue [3,6]. Different socioeconomic indicators were also found to be associated with mortality and cancer incidence in a US study [7]. In general, socioeconomic position is determined through such variables as occupation, education, income and wealth [3]. In the current study, we did not have information on income distribution. Our study illustrates the importance of gathering as much information as possible from a specific population if we wish to assess a potential confounding by area-level socioeconomic position in environmental epidemiology studies. In general, socioeconomic position is associated with individual health both at the individual and area level [6]. It is also important to highlight the potential impact of the spatial autocorrelation on the association estimates. Introducing the spatially lagged variable into the model allowed controlling for the presence of spatial autocorrelation.

Furthermore, in the same region, we discovered different sizes and directions of the associations, which underline the complexity of assessing the spatial correlation between exposure levels and socioeconomic patterns. This finding is consistent with that of other recent studies in Spain, in which it was established that environmental inequalities in spatially determined exposures may not always be great and may not always be negative in direction [41]. These findings may indicate that this is a national issue, rather than one typical only for a studied region. Further research is needed to clarify the uncertain relationship between socioeconomic indexes, especially in non-urban areas, where little is known about this issue.

The positive correlations found in mostly urban areas are in line with the findings in recent publications [26,42], in which populations with higher socioeconomic positions tended to be more exposed. This observation is in contrast with those of many other studies, which reported environmental disadvantages for groups with low socioeconomic status [1,3,20]. The inconsistent results across studies may be due to methodological differences or reflect different processes that underlie the relationship between pollution sources and socioeconomic factors [3].

Our analysis was performed at the census-tract scale, which is generally preferable to using zip codes [7]. In the urban area, census-tract scale is a fairly fine spatial scale and reflects neighborhood exposure. In sparsely populated rural areas, census-tract scale is on a large scale. Nevertheless, our study did not reflect small-scale variations related to the amount of traffic on the nearest road, which has been carried out in several investigations [26].

We used NO_2_ to represent the complex mixture of outdoor air pollution mixture; we employed NO_2_ as a surrogate for traffic-related exposure to ambient air pollutants, especially particulates, as has been done in previous studies [20,26]. NO_2_ was calculated from a LUR model [34], which was developed to assess precisely the risks of exposure, as have been suggested in numerous studies [43,44]. With this assessment, the mean levels for all the census tracts were below the annual limit of 40 μg/m^3^ recommended for NO_2_ by the World Health Organization air-quality guides [45] and established by European Directive 2008/50/CE [46].

Other studies have also used dispersion models [20]. The model used in the latter study included predictor variables, which have been used in other LUR models. It is very unlikely that these variables artificially induced a correlation, particularly in the urban areas. That model also included percentage of agricultural land cover, which can be inversely related to the variable used to split the analyses; however, land cover was categorized into continuous urban, discontinuous urban, agricultural, and industrial, and so in that case the variable percentage of industrial land could be used as a weighting variable. Moreover, we do not think that this type of relationship could have had an influence on the associations found in the present study.

One limitation of our study is that we evaluated outdoor exposures, not personal exposure. Hence differences in time activity patterns between different socioeconomic groups could not be accounted for. A French study suggested that while subjects in the least deprived neighborhoods in the suburbs experienced lower outdoor NO_2_ concentrations, their commuting exposures could be higher [23].

A further limitation is the combination of socioeconomic data for 2001 and pollution data for 2005. However, it is unlikely that both socioeconomic and pollution spatial patterns changed appreciably over the space of four years.

Associations between socioeconomic position and environmental exposure may be due to a variety of processes, such as housing prices and political decisions [3]. In the twentieth century, enormous growth in the population of the study region occurred owing to the construction of several large factories in the Avilés urban nucleus and its surroundings. In 1953, construction work began on the ENSIDESA factory—a large steel mill that is currently owned by Arcelor Mittal Heavy Steel Industry. More recently, other major companies in the area have included Saint Gobain Glass Ltd.; this company together with ENDASA (currently owned by Alcoa Inespal Aluminium Industry Ltd.), Asturian Zinc Industry Ltd., DuPont Industry, and Fertiberia Ltd. Have transformed Avilés into one of Spain’s main industrial centers (Additional file 3). This could explain the urban structure of the population studied, the great variability found in the rural areas, and the low correlation between pollution and educational level in this area.

Even though air pollution has become a major concern for its impact on health, and it may vary under different socioeconomic and demographic conditions, few studies in Spain have examined the distribution of air pollution levels by census tract, and related it to a socioeconomic index. With the present study, we were able to obtain maps of the pollution in Asturias and determine how the population is distributed with regard to demographic characteristics and different levels of NO_2_ exposure. From an epidemiological point of view, this study is important because socioeconomic characteristics may have an impact on the association between exposure levels and health outcomes.

## Conclusions

This study found associations between indicators of socioeconomic status and levels of air pollution in urban areas. It highlights that the strength and direction of the association between socioeconomic status and NO_2_ concentrations depends on the socioeconomic indicator used and the characteristics of the study area (urban/rural).

More research is needed in different scenarios to clarify the uncertain relationship between this factors and socioeconomic indexes, particularly in non-urban areas, where little investigation has been conducted on this topic.

## Abbreviations

NO2: Nitrogen dioxide; INMA: Environmental and Childhood; INE: National Statistical Institute; LUR: Land Use Regression; SE: Socioeconomic.

## Competing interests

The authors declare that they have no competing interests.

## Authors’ contribution

AFS performed the statistical analysis and drafted the manuscript. GH and AT conceived of the study, participated in its design and coordination, and revised the manuscript. All authors read and approved the final manuscript.

## Pre-publication history

The pre-publication history for this paper can be accessed here:

http://www.biomedcentral.com/1471-2458/13/71/prepub

## Supplementary Material

Additional file 1 Standard classification of occupations in Spain. Click here for file

Additional file 2 Standard classification of educational level in Spain. Click here for file

Additional file 3 **Study area (left); Roads, industrial facilities and land cover (upper right); Prediction map for NO**_**2 **_**(lower right).** Click here for file
