# Supplementary material for: Relationship between area-level socioeconomic characteristics and outdoor NO2 concentrations in rural and urban areas of northern Spain
Source: BMC Public Health. 2013 Jan 25;13:71. doi: 10.1186/1471-2458-13-71 (PMC3659019; doi:10.1186/1471-2458-13-71)
Supplement: Additional file 1 — Standard classification of occupations in Spain. [file 1471-2458-13-71-S1.doc]

Additional file 1. Standard classification of occupation in Spain

| Mark | Description |
| --- | --- |
| 0 | Under 16 years |
|  | With 16 years or older |
| 0.5  0  1  0.5  0  1 | *For those not occupied depending on their relationship with the activity:*  Unemployed people who had worked before  Unemployed seeking first work  Retired  Other pensioners  Other inactive  People living in groups |
| *2*  *1.5*  *1.5*  *2.5*  *1*  *3*  *3*  *2.5*  *2*  *3*  *2.5*  *2.5*  *2*  *1*  *2*  *2*  *1*  *2*  *2* | *For the employed, depending on socioeconomic status (SES), as follows:*  Agricultural employers with employees  Agricultural employers without employees  Members of agricultural cooperatives  Directors and heads of farms  Other farm workers  Professionals, technicians and equivalent which operate on their own, with or without employees  Non-agricultural employers with employees  Non-agricultural employers without employees  Non-agricultural cooperative members  Directors and managers of non-agricultural establishments, senior officials of government, autonomous communities and local government  Professional, technical and similar who perform work for others  Professionals in occupations unique to government  Rest of the administrative and commercial  Other personnel services  Boatswain and foremen non-agricultural establishments  Skilled workers and specialized non-agricultural establishments  Unskilled workers from non-agricultural establishments  Professional armed forces  Not classifiable by socio-economic condition |
