# Supplementary material for: Relationship between area-level socioeconomic characteristics and outdoor NO2 concentrations in rural and urban areas of northern Spain
Source: BMC Public Health. 2013 Jan 25;13:71. doi: 10.1186/1471-2458-13-71 (PMC3659019; doi:10.1186/1471-2458-13-71)
Supplement: Additional file 2 — Standard classification of educational level in Spain. [file 1471-2458-13-71-S2.doc]

Additional file 2. Standard classification of educational level in Spain

| Mark | Description |
| --- | --- |
| *0*  *1*  *2*  *2.5*  *3*  *3.5*  *4*  *4.5* | Illiterate (unable to read or write in any language)  No education (can read and write but was less than 5 years at school)  First degree (went to school 5 years or more but not complete primary or secondary)  Secondary  High school, medium grade vocational training, or equivalent industrial skilled working.  Higher grade vocational training, industrial Masters or equivalent, associate’s degree, Architecture and Engineering Techniques, 3 approved courses Degrees, Engineering or Architecture  Bachelor’s degree, Engineering or equivalent  Doctorate (PhD). |
