# Supplementary figures and images for: Relationship between area-level socioeconomic characteristics and outdoor NO2 concentrations in rural and urban areas of northern Spain
Source: BMC Public Health. 2013 Jan 25;13:71. doi: 10.1186/1471-2458-13-71 (PMC3659019; doi:10.1186/1471-2458-13-71)

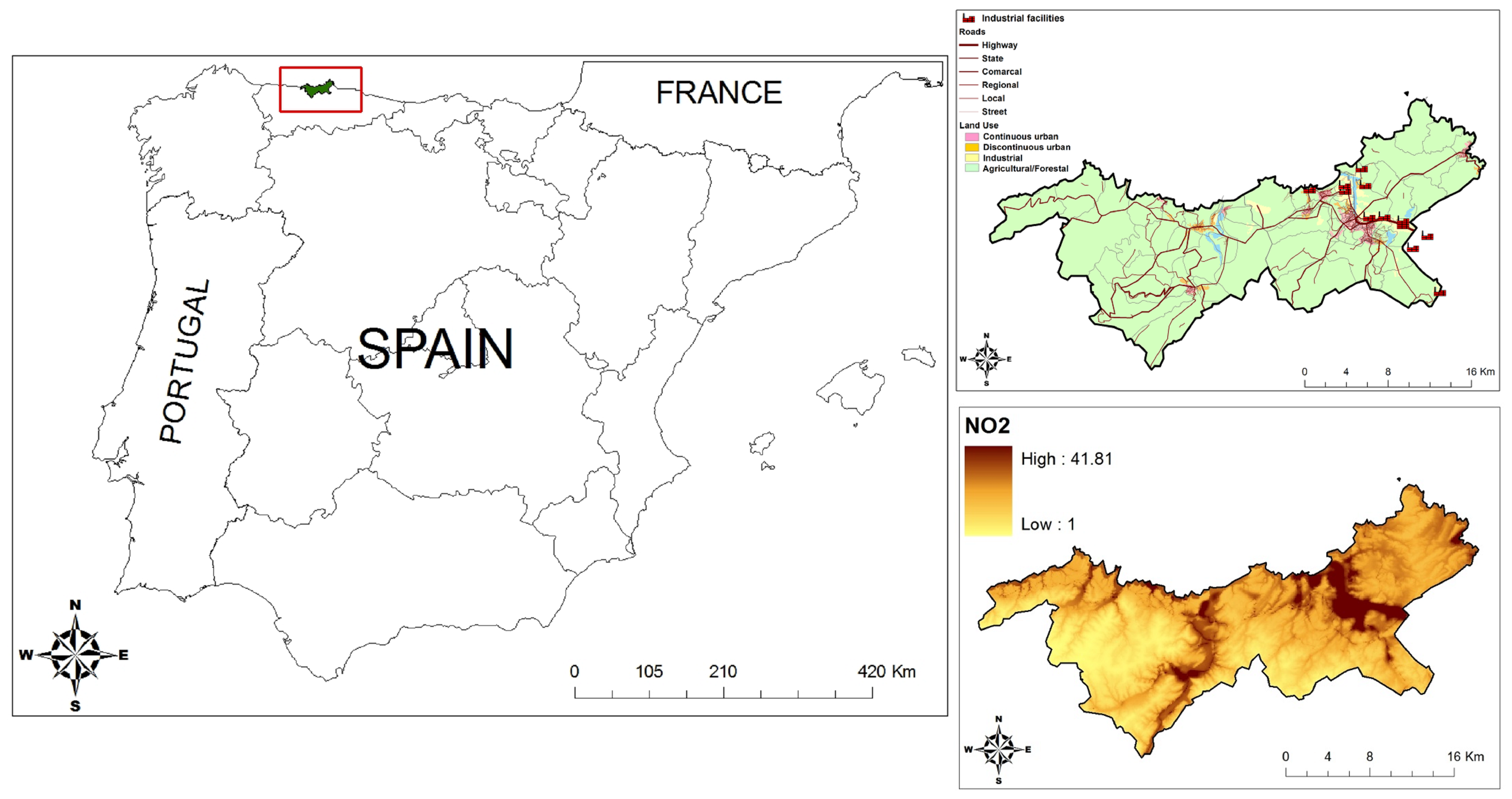

Supplement: Additional file 3 — Study area (left); Roads, industrial facilities and land cover (upper right); Prediction map for NO2 (lower right). [file 1471-2458-13-71-S3.tiff]
